# A dynamic lesion model for differentiation of malignant and benign pathologies

**DOI:** 10.1038/s41598-021-83095-2

**Published:** 2021-02-10

**Authors:** Weiguo Cao, Zhengrong Liang, Yongfeng Gao, Marc J. Pomeroy, Fangfang Han, Almas Abbasi, Perry J. Pickhardt

**Affiliations:** 1 Department of Radiology, State University of New York at Stony Brook, Stony Brook, NY USA; 2 Department of Biomedical Engineering, State University of New York at Stony Brook, Stony Brook, NY USA; 3 School of Biomedical Engineering, Southern Medical University, Guangzhou, 510515 Guangdong People’s Republic of China; 4 Department of Radiology, School of Medicine, University of Wisconsin, Madison, WI USA

**Keywords:** Cancer screening, Preclinical research, Biomedical engineering

## Abstract

Malignant lesions have a high tendency to invade their surrounding environment compared to benign ones. This paper proposes a dynamic lesion model and explores the 2nd order derivatives at each image voxel, which reflect the rate of change of image intensity, as a quantitative measure of the tendency. The 2nd order derivatives at each image voxel are usually represented by the Hessian matrix, but it is difficult to quantify a matrix field (or image) through the lesion space as a measure of the tendency. We conjecture that the three eigenvalues contain important information of the Hessian matrix and are chosen as the surrogate representation of the Hessian matrix. By treating the three eigenvalues as a vector, called Hessian vector, which is defined in a local coordinate formed by three orthogonal Hessian eigenvectors and further adapting the gray level occurrence computing method to extract the vector texture descriptors (or measures) from the Hessian vector, a quantitative presentation for the dynamic lesion model is completed. The vector texture descriptors were applied to differentiate malignant from benign lesions from two pathologically proven datasets: colon polyps and lung nodules. The classification results not only outperform four state-of-the-art methods but also three radiologist experts.

## Introduction

Malignant lesions have a high tendency to invade their surrounding environment compared to benign ones, thus the lesion growth rate has been widely used as a figure of merit (FOM) for computer-aided diagnosis (CADx) of the lesion, for example, of colon polyps^1,2^ and lung nodules^3,4^. However, this FOM requires at least two measurements over a time period of weeks or even months apart, incurring additional cost, patient stress and risk of delayed treatment.

On the other hand, numerous image features have been extracted from the lesion volume, followed by sophisticated feature selection and classification operations for the same task of CADx^5–8^. A typical example of a CADx pipeline takes a few steps of (1) localizing the lesion in a medical image [this step can be computerized, called computer-aided detection (CADe)]; (2) segmenting the lesion volume; (3) extracting features from the lesion volume, and (4) classifying the features for lesion diagnosis. By expanding the pipeline to include not only medical images but also genetic data and more, many successful applications have been reported in the field of radiomics^9–11^.

Recently, great efforts have been devoted to construct various convolutional neural network (CNN) architectures to deeply learn the features directly from the input medical images and simultaneously classify the learnt features for the same ultimate objective, i.e. CADx of lesions^12–15^. The efforts have been further devoted to expanding the CNN architectures to learn not only the medical images but also the textures and more^16,17^.

To our knowledge, both the above research endowers for sophisticated feature extraction and classification operations and the recent CNN-based deep machine learning architectures have not explicitly considered the clinical observations that malignant lesions have high tendency to invade their surrounding environment compared to benign ones^18–23^. This paper proposes a dynamic lesion model and explores the feasibility of explicitly considering the high invading tendency for the task of CADx of lesions.

The proposed dynamic lesion model is based on a conjecture that the high tendency is related to the rate of image intensity changing at each image voxel inside the lesion volume. The rate is mathematically described by the 2nd order derivative operation, usually represented by the Hessian matrix at each image voxel^24^. Thus, the dynamic lesion model is represented by a matrix field within the lesion space. Extracting quantitative descriptors from the matrix field or matrix image to measure the tendency is mathematically challenging. To relieve this mathematical challenge, we have another conjecture below.

Based on the recent report that the eigenvalues and the eigenvectors of the Hessian matrix are mathematically related to each other^25^, we conjecture that the three eigenvalues contain most, if not all, the information of the Hessian matrix and, therefore, the three eigenvalues are chosen as the surrogate representation of the Hessian matrix. By treating the three eigenvalues as a vector, called Hessian vector hereafter, described by its two angular variables and one magnitude variable at each image voxel, we can obtain a vector image through the lesion volume. By adapting the well-known gray level co-occurrence (GLCM) computing method^7,26^ to extract texture measures (or descriptors) from the vector image, followed by classification of the extracted vector texture descriptors, a quantitative description for the dynamic lesion model is completed.

Experimental results from pathologically proven lesion datasets demonstrate the superiority of the dynamic lesion model over state-of-the-art classification methods^26–29^. Particularly, the demonstration of outperformance of the presented dynamic lesion model over three experienced radiologists indicate the great potential of model-based, task-driven, artificial intelligence (AI)-enabled CADx, which not only learns the experience that experts have learned, but also knows the task that experts are heading to. A similar demonstration of the great potential is the task-driven, AI-enabled AlphaGo^30^, which can outperform experts because it not only learns how to play the game from the experts, but also knows the task of occupying the largest space when taking each step further, while experts may not be able to achieve that task at every step.

The remainder of this paper is organized as follows. “Methods” describes the dynamic lesion model and the model representation by Hessian matrix and its eigenvalue vector at each image voxel, followed by the presentation of GLCM computation for extraction of the vector texture descriptors from the Hessian vector image. “Experiments and results” presents the experimental outcomes from two pathologically proven datasets, colon polyps and lung nodules, for the task of differentiating malignant from benign lesions with comparison to four existing state-of-the-art lesion classification CADx methods and three radiologist experts. Lastly, discussions and conclusions are drawn in “Conclusions and discussions”.

## Methods

As introduced above, this paper proposes a novel dynamic lesion model to perform the CADx task. Inspired by the clinical observation of different invasion properties for different lesion types, the model is deliberately designed to express the invading tendency using Hessian Matrix representation. In this section, we will describe the dynamic model in detail.

### Dynamic lesion model and hessian matrix representation

Our proposed dynamic lesion model is based on the observations that a malignant lesion has a high tendency to invade its surrounding environment compared to a benign one^20,21^. We hypothesize that the tendency for lesions to invade their surrounding environment is related to the rate of change of the image intensity values at each image voxel. The proposed method uses a Hessian matrix representation of the 2nd order derivative to model these changes in intensity.

The rate of the change is mathematically expressed by the 2nd order derivative operator of a scalar function or intensity image $I = I\left( {x,y,z} \right)$ in three-dimensional (3D) space and could be represented by the Hessian matrix as follows^24^:

1 $$ {\mathbf{H}}\left( {x,y,z} \right) = \left[ {\begin{array}{*{20}c} {I_{xx} } & {I_{xy} } & {I_{xz} } \\ {I_{xy} } & {I_{yy} } & {I_{yz} } \\ {I_{xz} } & {I_{yz} } & {I_{zz} } \\ \end{array} } \right], $$

where *I*_*xx*_, *I*_*xy*_, *I*_*xz*_, *I*_*yy*_, *I*_*yz*_, and *I*_*zz*_ are the 2nd order derivatives of $I\left( {x,y,z} \right)$. To compute these partial derivatives of the intensity image, we use the well-established Deriche filters with parameter $\alpha = 1$ as a default in the article^31^. The lesion model is then represented by a matrix at each image voxel through the lesion space, i.e. a matrix field or matrix image. Constructing image feature measures or descriptors from a matrix field for the task of CADx of lesions is difficult. To mitigate this difficulty, we adapt the traditional analysis of decomposing the Hessian matrix at each image voxel location.

### Hessian eigenvalues as the surrogate representation of hessian matrix

Traditionally, Hessian matrix is frequently utilized to describe local image geometries, such as edges and corners in image processing^32,33^. Because of the 2nd order derivative operation on a scalar function, Hessian matrix reflects the rate of the scalar function changing, which describes not only the local geometric (shape) properties but also the local moving tendency. While Hessian matrix contains very useful local geometric and dynamic information of a lesion in a medical image, it is very challenging to directly utilize it to construct image feature descriptors for the task of CADx of lesions. To combat this challenge, we adapt the traditional approach of decomposing the Hessian matrix as follows^24^:

2 $$ \left[ {\begin{array}{*{20}c} {I_{xx} } & {I_{xy} } & {I_{xz} } \\ {I_{xy} } & {I_{yy} } & {I_{yz} } \\ {I_{xz} } & {I_{yz} } & {I_{zz} } \\ \end{array} } \right]\left( {\begin{array}{*{20}c} {{\varvec{v}}_{1} } & {{\varvec{v}}_{2} } & {{\varvec{v}}_{3} } \\ \end{array} } \right) = \left( {\begin{array}{*{20}c} {{\varvec{v}}_{1} } & {{\varvec{v}}_{2} } & {{\varvec{v}}_{3} } \\ \end{array} } \right)\left[ {\begin{array}{*{20}c} {\lambda_{1} } & 0 & 0 \\ 0 & {\lambda_{2} } & 0 \\ 0 & 0 & {\lambda_{3} } \\ \end{array} } \right], $$

where $\lambda_{1} \ge \lambda_{2} \ge \lambda_{3}$ are three eigenvalues, ${\varvec{v}}_{1}$,${\varvec{v}}_{2}$*,* and ${\varvec{v}}_{3}$ are their corresponding eigenvectors, respectively, which are all orthogonal with each other and form three new basis in the 3D Euclidean space.

Based on the recent report that the eigenvalues and the eigenvectors are mathematically related to each other^25^, we can choose either the three eigenvalues or the three eigenvectors to construct image feature descriptors. Since the three eigenvalues contain fewer variables than the three eigenvectors and have advantages in computation, the eigenvalues would be a better choice.

Furthermore, the three eigenvalues can be viewed as the projections of a vector on the three orthogonal eigenvectors, respectively, and the vector contains not only the strength of motion tendency (i.e. the vector magnitude), but also the direction of motion tendency (the vector orientation). Thus, we have another conjecture that the vector, made from the three eigenvalues and called Hessian vector hereafter, is a surrogate representation of the Hessian matrix.

Based on the two conjectures above, our dynamic lesion model is then represented by the Hessian vector. For the CADx task, we will turn to how to extract quantitative measures about the lesion tendency from the vector field.

### Vector-based co-occurrence matrix or vector texture image

**Figure 1:**
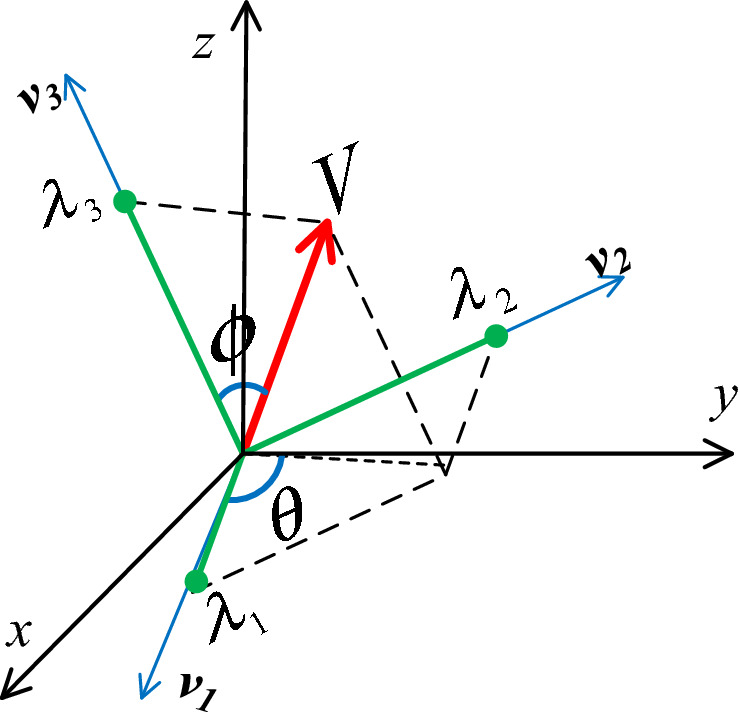
Diagram showing the azimuth and polar angles $\theta$ and $\emptyset$ respectively from the Hessian vector $V\left( {x,y,z} \right) = \left( {\lambda_{1} ,\lambda_{2} ,\lambda_{3} } \right)$.

From the decomposition of Hessian matrix, one image voxel in volumetric data could produce three eigenvalues which could be formed as a local vector (or Hessian vector), i.e. $\left( {\lambda_{1} ,\lambda_{2} ,\lambda_{3} } \right)$. This 3D vector image could be represented at each image voxel by:

3 $$ {\varvec{V}}\left( {x,y,z} \right) = \left( {\lambda_{1} \left( {x,y,z} \right),\lambda_{2} \left( {x,y,z} \right),\lambda_{3} \left( {x,y,z} \right)} \right), $$

4 $$ \lambda_{{\text{m}}} < \lambda_{{\text{i}}} \left( {x,y,z} \right) < \lambda_{{\text{M}}} , $$

where $\left( {x,y,z} \right)$ is the coordinate in the original image $I\left( {x,y,z} \right)$, and the values $\lambda_{{\text{m}}}$ and $\lambda_{{\text{M}}}$ are the minimum and maximum eigenvalues among all the image voxels. Thus, each image voxel inside the Hessian vector field is specified by its three values of $\left( {\lambda_{1} ,\lambda_{2} ,\lambda_{3} } \right)$ of Eq. (3) within the range of Eq. (4). Figure 1 illustrates that ${\varvec{V}}\left( {x,y,z} \right)$ should be a Hessian vector in a new local 3D Euclidean space, defined by its three orthogonal eigenvectors, i.e. ${\varvec{v}}_{1}$***, ***${\varvec{v}}_{2}$*,* and ${\varvec{v}}_{3}$.

As a vector, $V\left( {x,y,z} \right)$ could be expressed by the spherical coordinates as:

5 $$ {\varvec{V}}\left( {x,y,z} \right) = \left( {\lambda_{1} ,\lambda_{2} ,\lambda_{3} } \right) = \left| \lambda \right|\left( {cos\theta cos\emptyset ,sin\theta cos\emptyset ,sin\emptyset } \right), $$

where |$\lambda$| is the magnitude, $\theta$ and $\emptyset$ are two angles representing the vector direction as shown in Fig. 1. For simplicity in presentation, |$\lambda$|= $\lambda$ will be assumed hereafter. The Hessian vector of Eq. (5) is defined by the three variables as follows:

6 $$ \lambda = \sqrt {\left( {\lambda_{1} } \right)^{2} + \left( {\lambda_{2} } \right)^{2} + \left( {\lambda_{3} } \right)^{2} } , $$

7 $$ \theta = \left\{ {\begin{array}{*{20}c} {\cos^{ - 1} \left( {{\raise0.7ex\hbox{${\lambda_{1} }$} \!\mathord{\left/ {\vphantom {{\lambda_{1} } {\sqrt {\left( {\lambda_{1} } \right)^{2} + \left( {\lambda_{2} } \right)^{2} } }}}\right.\kern-\nulldelimiterspace} \!\lower0.7ex\hbox{${\sqrt {\left( {\lambda_{1} } \right)^{2} + \left( {\lambda_{2} } \right)^{2} } }$}}} \right)} & {\lambda_{2} \ge 0} \\ {\pi + \cos^{ - 1} \left( {{\raise0.7ex\hbox{${\lambda_{1} }$} \!\mathord{\left/ {\vphantom {{\lambda_{1} } {\sqrt {\left( {\lambda_{1} } \right)^{2} + \left( {\lambda_{2} } \right)^{2} } }}}\right.\kern-\nulldelimiterspace} \!\lower0.7ex\hbox{${\sqrt {\left( {\lambda_{1} } \right)^{2} + \left( {\lambda_{2} } \right)^{2} } }$}}} \right)} & {\lambda_{2} < 0,} \\ \end{array} } \right. $$

8 $$ \emptyset = \cos^{ - 1} \left( {{\raise0.7ex\hbox{${\lambda_{3} }$} \!\mathord{\left/ {\vphantom {{\lambda_{3} } {\sqrt {\left( {\lambda_{1} } \right)^{2} + \left( {\lambda_{2} } \right)^{2} + \left( {\lambda_{3} } \right)^{2} } }}}\right.\kern-\nulldelimiterspace} \!\lower0.7ex\hbox{${\sqrt {\left( {\lambda_{1} } \right)^{2} + \left( {\lambda_{2} } \right)^{2} + \left( {\lambda_{3} } \right)^{2} } }$}}} \right). $$

Since $\lambda$, $\theta$ and $\emptyset$ are three geometric measures, they are independent with any coordinates even if they are expressed by $\lambda_{1} ,\lambda_{2} ,{\text{and }}\lambda_{3}$ which are three functions expressed by ($x,y,z$). So $\lambda$, $\theta$, and $\emptyset$ are three very stable local metrics, which are more suitable to describe local properties in lesions.

Given the Hessian vector images of Eqs. (5)–(8) from the acquired intensity image, $I = I\left( {x,y,z} \right)$, our task is to find or develop a computing method to extract quantitative measures from these vector images.

The magnitude (${\uplambda }$), azimuth angle ($\theta$) and polar angle ($\emptyset$) form another equivalent Hessian vector of Eq. (3) which produces the vector image, represented at each image voxel by:

9 $$ {\varvec{V}}\left( {x,y,z} \right) = \left( {\lambda ,\theta ,\emptyset } \right), $$

10 $$ \lambda_{{\text{m}}} < \lambda < \lambda_{{\text{M}}} ,\quad 0 \le \theta \le 2\pi ,\quad 0 \le \emptyset \le \pi , $$

where the minimum value $\lambda_{{\text{m}}}$ and the maximum value $\lambda_{{\text{M}}}$ are determined by Eq. (4), given the acquired image $I\left( {x,y,z} \right)$. Thus, each image voxel inside the Hessian vector field is specified by its three values of $\left( {\lambda ,\theta ,\emptyset } \right)$ of Eq. (9) within the ranges of Eq. (10), respectively.

Inspired by the well-known GLCM texture descriptor for CADx of lesions^7,26^, our task here is to find an adequate number of gray levels for each of the three ranges of Eq. (10). Then, GLCM measures can be computed from the vector image of Eq. (9).

#### *Digitalization of the three variables in their ranges of Eq. (**10**), respectively*

Let $Q^{a}$ and $Q^{b}$ denote the numbers of gray levels of $\theta$ in the range [0,$2\pi ]$ and $\phi$ in the range [0,$\pi$], respectively. The digitalization of the angular variables $\theta$ and $\emptyset$ in the vector image domain is given by:

11 $$  \Theta  = \left\{ {\begin{array}{*{20}c}    {\left\lfloor {{\raise0.7ex\hbox{${\left( {Q^{a}  \cdot \theta } \right)}$} \!\mathord{\left/ {\vphantom {{\left( {Q^{a}  \cdot \theta } \right)} {2\pi }}}\right.\kern-\nulldelimiterspace} \!\lower0.7ex\hbox{${2\pi }$}}} \right\rfloor ~} & {\theta  \ne 2\pi }  \\    {Q^{a}  - 1} & {\theta  = 2\pi ,}  \\   \end{array} } \right.  $$

12 $$ \Phi  = \left\{ {\begin{array}{*{20}c}    {\left\lfloor {{\raise0.7ex\hbox{${\left( {Q^{b}  \cdot \phi } \right)}$} \!\mathord{\left/ {\vphantom {{\left( {Q^{b}  \cdot \phi } \right)} \pi }}\right.\kern-\nulldelimiterspace} \!\lower0.7ex\hbox{$\pi $}}} \right\rfloor } & {\phi  \ne \pi }  \\    {Q^{b}  - 1} & {\phi  = \pi ,}  \\   \end{array} } \right. $$

where ⌊*X*⌋ is defined as the infimum of *X*^28^.

Due to the scale difference between the angular variables ($\theta$, $\emptyset$) and the magnitude variable (${\uplambda }$), we introduce a scale mapping parameter ${\uptau }$, where ${\uptau }$ is an integer. Let $Q^{c}$ denote the number of gray levels of $\rho$ in the range [$\rho_{m} ,\rho_{M}$] and $\Delta \rho = \rho_{M} - \rho_{m}$. The digitalization of the vector magnitude $\rho$ is given by:

13a $$ \rho = \sqrt[\tau ]{{\uplambda }}, $$

13b $$  \rho  = \left\{ {\begin{array}{*{20}c}    {\left\lfloor {{\raise0.7ex\hbox{${\left( {\left( {\rho  - \rho _{m} } \right) \cdot Q^{c} } \right)}$} \!\mathord{\left/ {\vphantom {{\left( {\left( {\rho  - \rho _{m} } \right) \cdot Q^{c} } \right)} {\Delta \rho }}}\right.\kern-\nulldelimiterspace} \!\lower0.7ex\hbox{${\Delta \rho }$}}} \right\rfloor } & {\rho  \ne \rho _{M} }  \\    {Q^{c}  - 1} & {\rho  = \rho _{M} .}  \\   \end{array} } \right. $$

Thus, we obtain the gray level vector image from the acquired intensity image, $I = I\left( {x,y,z} \right)$, as follows:

14 $$ \user2{\rm T} = \left( {{\varvec{\rho}},{\Theta } ,{\Phi }} \right). $$

Our next task is to compute the GLCM-alike measures from this gray level vector image.

#### Computation of vector-based co-occurrence matrix

**Figure 2:**
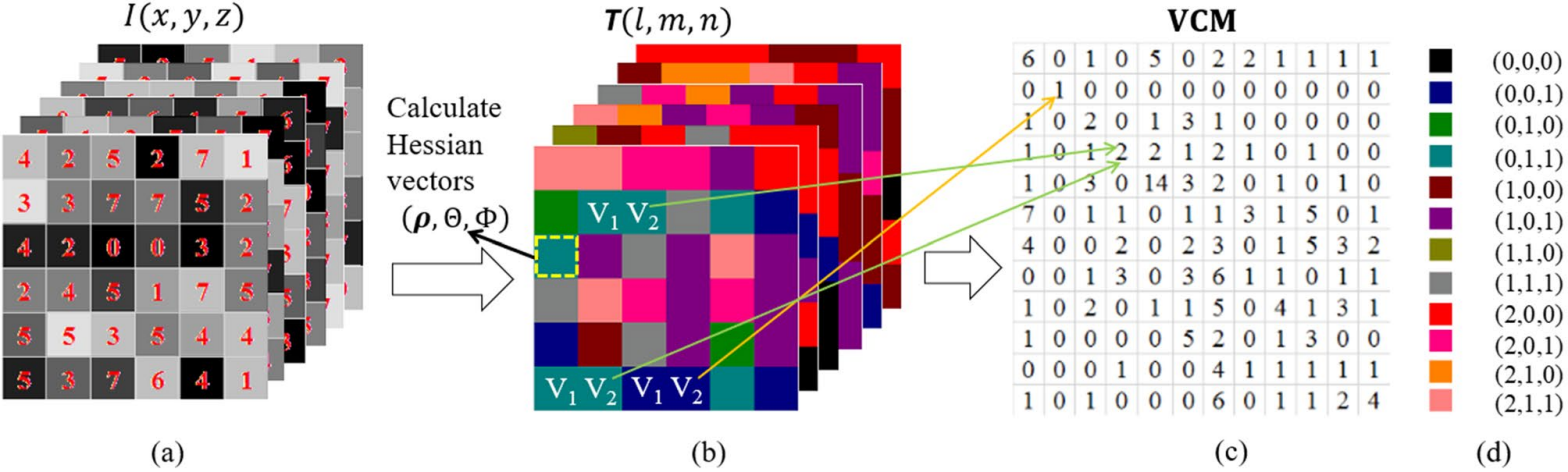
An illustration of vector-based **CM** calculation. (**a**) Illustration of one 6 × 6 × 6 volumetric data with 8 Gy levels as an example for illustration purpose. (**b**) Visualization of Hessian vector-based 3D image where every element is a Hessian vector and $Q^{c} = 3$, $Q^{a} = Q^{b} = 2$ are chosen as an example. (**c**) The **VCM** of (**b**) with offset (or direction) (0, 1, 0) where the total Hessian vector number is equal to 3 × 2 × 2 = 12. (**d**) The color maps used by 12 vectors in (**b**).

Given the above gray level vector image of Eq. (14), we present in this section a method which utilizes derivative-based vector to define a new type of co-occurrence matrix (**CM**), called vector-based **CM** or **VCM** and expressed as:

15 $$ {\mathbf{VCM}}_{{V_{1} ,V_{2} }} \left( {\Delta l,\Delta m,\Delta n} \right) = \mathop \sum \limits_{n = 1}^{N} \mathop \sum \limits_{m = 1}^{M} \mathop \sum \limits_{l = 1}^{L} \left\{ {\begin{array}{*{20}c} 1 & {\begin{array}{*{20}c} {{\varvec{T}}\left( {l,m,n} \right) = {\varvec{V}}_{1} \& \& } \\ {{\varvec{T}}\left( {l + \Delta l,m + \Delta m,n + \Delta n} \right) = {\varvec{V}}_{2} } \\ \end{array} } \\ 0 & {otherwise} \\ \end{array} } \right., $$

where ${\varvec{T}}\left( {l,m,n} \right)$ represents the digitalized gray level vector images in 3D space, (L,M,N) indicate the 3D volume size, ${\varvec{V}}_{1}$ and ${\varvec{V}}_{2}$ are a vector pair in ${\varvec{T}}\left( {l,m,n} \right)$, and $\left( {\Delta l,\Delta m,\Delta n} \right)$ is the offset from ${\varvec{T}}\left( {l,m,n} \right)$ to ${\varvec{T}}\left( {l + \Delta l,m + \Delta m,n + \Delta n} \right)$, indicating a direction. Figure 2 illustrates the calculation of Eq. (15).

How to find all offsets around one concerned voxel is one of several key steps in the calculation of **VCM**. It is well known that the nearest neighbors have the closest relevance to the concerned voxel in topology and statistics. Therefore, the 26 nearest neighbor voxels around the concerned voxel are chosen as our candidates to obtain the offsets, i.e. $\left( {\Delta l,\Delta m,\Delta n} \right)$, in Eq. (15). These offsets can be viewed as the data sampling in the 3D vector image domain along the 26 directions defined by the 26 nearest neighbors^34^.

By the **VCM** definition of Eq. (15), the two **VCM**s with one offset and its inverse offset (or inverse direction) are symmetrical so that half of the 26 nearest neighbor voxels are redundant and should be discarded. Therefore, 13 offsets are retained while their inverse directions are removed. The retained 13 offsets for the **VCM** calculation are denoted as: (0, 0, 1), (0, 1, 0), (1, 0, 0), (0, 1, 1), (1, 0, 1), (1, 1, 0), (− 1, 1, 0), (0, 1, − 1), (1, 0, − 1), (1, 1, 1), (− 1, 1, 1), (1, 1, − 1) and (− 1, 1, − 1). Thus, using the **VCM** definition of Eq. (15), we will compute 13 **VCM**s with 13 offsets through a lesion volume. Each of these 13 **VCM**s is called vector texture pattern or image hereafter.

#### Extraction of vector texture measures from VCMs

By adapting the 28 texture measure definitions from each offset^35^, we will extract a total of 364 (= 13 × 28) vector texture measures per lesion, i.e. 28 measures per direction over 13 offsets. Since these 364 texture measures are derived from a 3D lesion and the redundancy from the 13 reverse offsets is not included, they will be called vector texture features (VTFs) hereafter. Classifying these VTFs and evaluating the classification outcomes are presented in the following section.

### Classification of the vector texture features

This study proposed a dynamic lesion model to differentiate malignant from benign pathologies. The evaluation of the lesion model is based on two pathology proven datasets, i.e. polyp and nodule datasets. The polyp data acquisition is approved by the Institutional Review Board (IRB) from University of Wisconsin. The nodule data acquisition is approved by the IRB of Stony Brook University. The use of the datasets for the evaluation is approved by the IRB of Stony Brook University. All methods in this study were performed in accordance with the relevant guidelines and regulations.

Once the VTFs are computed for each lesion’s gray level vector image of Eq. (14), which is the 2nd order derivative representation of the original image of $I\left( {x,y,z} \right)$, many classifiers can be used to classify these vector texture features, as presented in the introduction section above. To focus on the demonstration of the impact of the proposed lesion model with the associated VTFs extraction strategy for the task of CADx of lesions, we adapt the R package “random forest (RF)” to perform the classification^36,37^, as it has shown effectiveness in our previous classification experiments^7,34^. Exploring new machine learning classification methods on the VTFs is beyond the scope of this work.

Due to the limited sample size of pathologically proven medical image datasets, the RF classifier (and all machine learning algorithms) are susceptible to bias from the input data of limited sample size, depending on how it is divided into training and testing data subsets. To reduce this bias effect, 100 randomized groups of lesions are generated, and each group is divided into training and testing sets. The division is made such that there is an equal number of each class (or lesion label) in both the training and testing sets.

We employ a feature selection algorithm from the R package that first reads in the entire list of the vector texture features and generates an importance value for each variable in the feature set based on the GINI index^36^. We use a forward step feature selection (FSFS) method^7,38^ to perform lesion classification, where the features are added iteratively to that algorithm in decreasing importance order, such that the best performing features are added first to optimize performance. This procedure is repeated for each of the 100 randomized groups of testing and training datasets. The results are evaluated for each group and for each number of features based on the area under the curve (AUC) of receiver operating characteristics curve (ROC) with average over the 100 groups. Results of the above 100 repeated two-fold cross validation experiments are shown in the following section.

## Experiments and results

In this section, we first describe the clinical datasets, which will be used to test the proposed lesion model, and evaluate two parameters associated with the digitalization of gray levels and the scale mapping of the magnitude of the Hessian vector to obtain some preliminary results for further experiments. Then lesion classification is performed on the extracted vector texture features or VTFs. At the end, we compare our classification results with four existing CADx methods and three radiologist experts.

### Polyp and nodule datasets

#### Polyp dataset

A total of 59 patients including 51% males and 49% females, who were scheduled for clinical colonography examination, were recruited to this study under informed consent after approval by the IRB. Their ages range from 45.9 to 91.6 years old (mean age of 66.5 years old). The patients were scanned by a routine clinical non-contrast CT colonography (CTC) scanning protocol covering the entire abdomen volume prior to the clinical colonography examination. Tube voltage was 120 kVp and dose was determined by automatic exposure control. A total of 63 polyp masses were found and resected by the clinical examination. The pathological reports indicate 31 benign and 32 malignant polyps. The size of the polyp masses ranges from 3 to 8 cm (mean of 4.2 cm). In the group of benign, four sub-categories are recorded from the pathology reports, they are Serrated Adenoma (3 cases), Tubular Adenoma (2 cases), tubulovillous adenoma (21 cases), Villous Adenoma (5 cases). In the category of malignant, it is Adenocarcinoma only (32 cases). Each abdominal CTC image volume consists of more than 400 image slices, each image slice has an array size of 512 × 512, and each image element or voxel is nearly cubic with edge size of 1 mm. The contour of each polyp image slice inside the CTC abdominal image volume was manually delineated by radiological experts on a slice-by-slice manner using a semi-automated segmentation algorithm.

#### Pulmonary nodule dataset

A total of 66 patients including 52% males and 48% females, who were scheduled for CT-guided lung nodule needle biopsy, were engaged in this study under informed consent after approval by the IRB. The average age of the patients is 69.5 years old, ranging from 33 to 91 years old. A total of 68 lung nodules with 20 benign and 48 malignant were biopsied under the CT scanning with 120 kVp tube voltage and automatic exposure control. The diameter of these nodules ranges from 0.91 to 13.08 cm (mean size of 3.15 cm). Each CT scan covers a portion of the patient entire chest volume, resulting in 100–200 image slices of 512 × 512 array size, and each image voxel is nearly cubic with edge size of 1 mm. The border of each nodule image slice inside the volumetric patient CT scan was also drawn by experts on a slice-by-slice manner using a semiautomated segmentation algorithm.

### Experimental results from polyp dataset

To calculate the **VCM**s of polyps, the nearest 26 neighbors around the concerned voxel were used to determine the 13 independent offsets or directions which were described in “Vector-based Co-occurrence Matrix or Vector Texture Image”. According to the digitalization scheme of Eqs. (11)–(13b), the total gray levels are equal to $Q^{a} \times Q^{b} \times Q^{c}$. To study the number of total gray levels (i.e. $Q^{a} ,Q^{b} ,Q^{c}$) as a parameter in calculating the **VCM**, fifteen different total gray levels were considered in our experiments, and the parameter varies in the range of {16, 24, 32, 40, 48, 56, 64, 72, 80, 88, 96, 104, 112, 120, 128}. After integer factorization, these 15 total gray levels will generate 235 different combinations of $Q^{a} ,Q^{b} ,{\text{ and}} Q^{c}$ as listed in the Appendix of this paper. These combinations will produce 235 feature sets which will be fed to the RF classifier as training and testing datasets. The parameters which produced the best outcome were then chosen as the total gray levels for the digitalization scheme. The parameters of (2, 2, 32) and (2, 8, 6) for ($Q^{a} ,Q^{b} ,Q^{c}$) were chosen in the experiments for classification of polyp and nodule datasets respectively.

The above trial-and-error empirical method for parameter determination was also applied to determine the parameter ${\uptau }$ for the scale mapping of the vector magnitude in Eq. (13a). We tested this parameter in the range of 1, 2, …, 6. The range of 2, 3, 4, 5 is reasonable choice. ${\uptau } = 5$ was chosen in the experiments for classification of our polyps and ${\uptau } = 2$ is selected for our pulmonary nodules.

Once the two parameters were determined, the following steps are taken to generate the final classification results. For the 63 polyps of limited sample size, including 31 benign and 32 malignant ones, we designed three testing schemes, aiming to perform a thorough investigation. We performed the two-fold cross validation as described in the method section above. To obtain robust results, 100 observation groups were randomly selected from the dataset. For each observation group, we selected 31 polyps as the training set and the remaining 32 polyps as the testing set. The experimental set up is presented in the first row of Table 1. After classification, we evaluate the results with four widely used metrics, i.e. AUC, Accuracy (Acc), Sensitivity (Sen), and Specificity (Spe). The classification results, in terms of average and standard deviation from the 100 observation groups, are shown in the first row of Table 2.

**Table 1:** The training and testing scheme for the polyp and lung nodule dataset.

	Training			Testing/prediction		
	Benign	Malignant	Observation group	Benign	Malignant	Observation group
Polyps	15	16	100	16	16	100
Lung nodules	10	24	100	10	24	100

**Table 2:** The classification results of the polyp and lung nodule dataset.

	AUC	Acc	Sen	Spe
Polyps	0.986 ± 0.012	0.959 ± 0.026	0.939 ± 0.055	0.980 ± 0.034
Lung nodules	0.861 ± 0.061	0.841 ± 0.052	0.863 ± 0.087	0.786 ± 0.103

### Experimental results from nodule dataset

**VCM** and VTF calculations of the pulmonary nodules share the same steps as the **VCM** and VTF calculations of the colon polyps, i.e. going through the 13 independent offsets or directions and performing the digitalization scheme to obtain the 235 different combinations of $Q^{a} ,Q^{b} , {\text{and }}Q^{c}$ for the corresponding 235 features. By inputting these 235 features to the RF package and going through the GINI-based feature sorting and the FSFS-based feature selection^7,38^, the two parameters (one is the total number of gray levels and the other is the scale mapping of the vector magnitude) will be determined. Once the two parameters were determined, the following procedure is taken to generate the final classification results, similar to the procedure in generating the final classification results of the polyps.

In this classification, the 68 nodules, including 20 benign and 48 malignant, were stochastically divided into 100 groups of training and testing datasets with twofold cross validation as illustrated in the second row of Table 1. The final classification results, in terms of the average and standard deviation over the 100 groups, are shown in the second row of Table 2. Compared to the polyp data results of the first row in Table 1, it is very interesting to note the gap of 8% between the sensitivity and specificity in the very unbalanced nodule dataset (of 20/48) vs. the gap of 4% in the nearly balanced polyp dataset (of 31/32). This may reflect the robustness of the lesion model for unbalanced datasets.

### Comparison to other CADx methods

For reference on how well our proposed dynamic lesion model performs, we compare our results to some well-known texture extraction and classification methods and a state-of-the-art CNN deep learning architecture on both the polyp and the nodule datasets as follows.

- Haralick texture method^26^ with extended measures (eHM)^7,35^. The Haralick method is widely cited for the GLCM texture descriptor in the field of CADx.
- HoG3D^27^—this method counts the occurrences of gradient orientation (i.e. only the angular variables of a vector) in some cropped portions of the original intensity image and generates some histograms which are joined to form gradient features.
- CoLIAGe (co-occurrence of local anisotropic gradient orientation^28^—this mothed also employs the gradient angles to extract the entropy of every local patch (or a local voxel group) to form texture features by two joint histograms.
- VGG16^29^—it is a widely cited CNN deep learning method. It was implemented by the following architecture: total of 20 salient slices were extracted from each lesion volume and inputted to the VGG16 pipeline for lesion feature extraction and classification.

**Figure 3:**
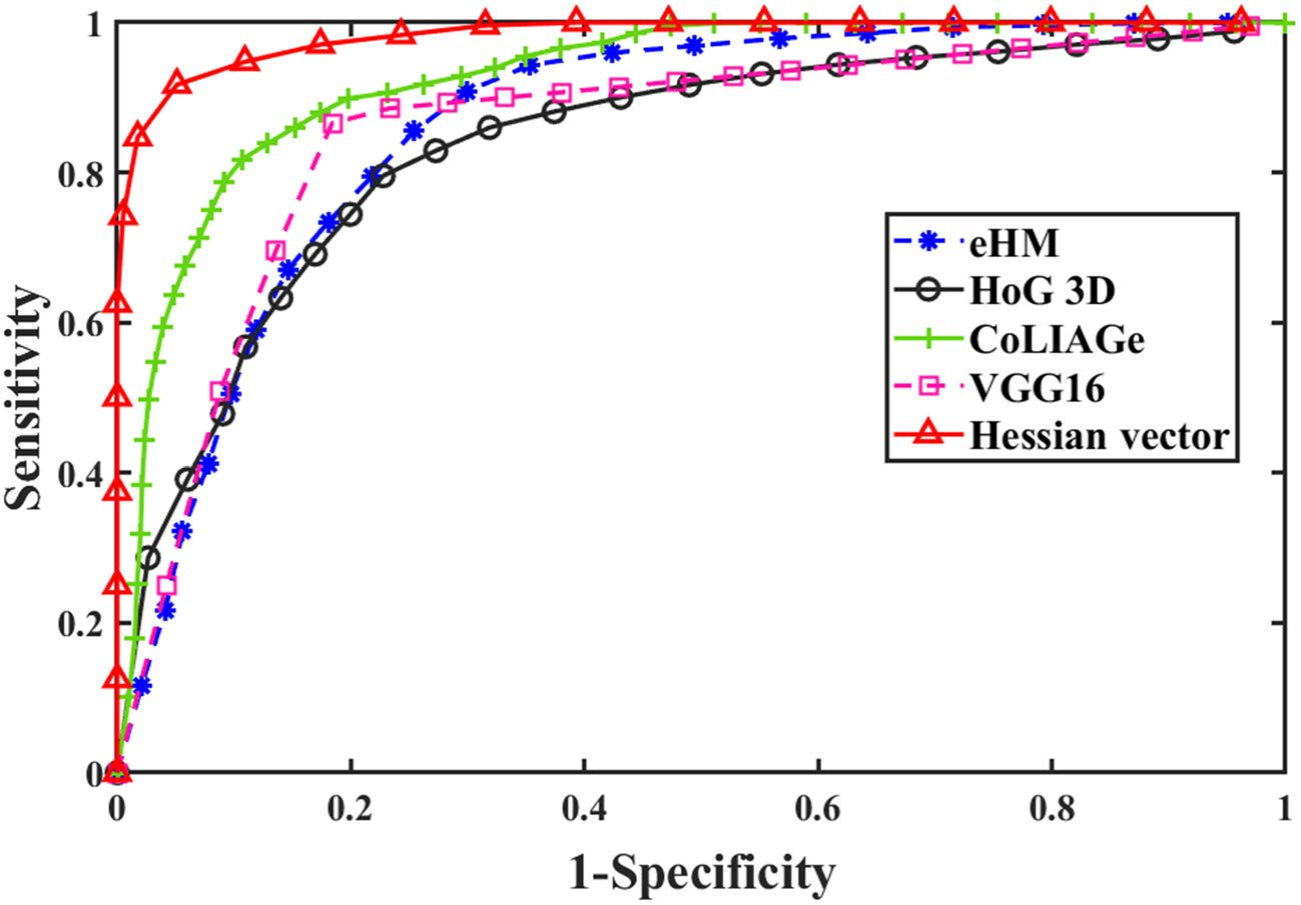
The ROC curves presented for each comparative method and our proposed lesion model over the 63 polyps with twofold cross validation.

Figure 3 shows the ROC curves of the Hessian vector representation of our lesion model with comparison to the above four CADx methods over the 63 polyps. These ROC curves demonstrate that VTF based polyp classification is the best one among the four state-of-art methods.

In addition to the visual assessment via ROC plots, we further computed the four evaluation metrics. Table 3 shows the comparison results among these four different methods and our proposed lesion model over the 63 colon polyps, where the same RF classifier was used for eHM, HoG3D and CoLIAGe. Our proposed lesion model performed the best. We can see quite clearly that when compared to the eHM, the *Hessian vector* derived texture features outperformed the Haralick texture features with an AUC of 0.982 against 0.876. Compared against the gradient-based features of HoG3D and CoLIAGe, our proposed vector-based texture features improved the performance substantially over the HoG3D features (AUC = 0.804) and the CoLIAGe texture features (AUC = 0.923). The gain of our lesion model is also substantially higher over the VGG-16 outcome (AUC = 0.833).

**Table 3:** AUC, accuracy, sensitivity, and specificity values over the 63 colon polyps from the comparative methods and our proposed lesion model.

	AUC	Acc	Sen	Spe
eHM	0.876	0.807	0.858	0.757
HoG3D	0.804	0.713	0.726	0.700
CoLIAGe	0.923	0.836	0.839	0.833
VGG16	0.833	0.740	0.709	0.771
Hessian vector	0.986	0.959	0.939	0.98

**Figure 4:**
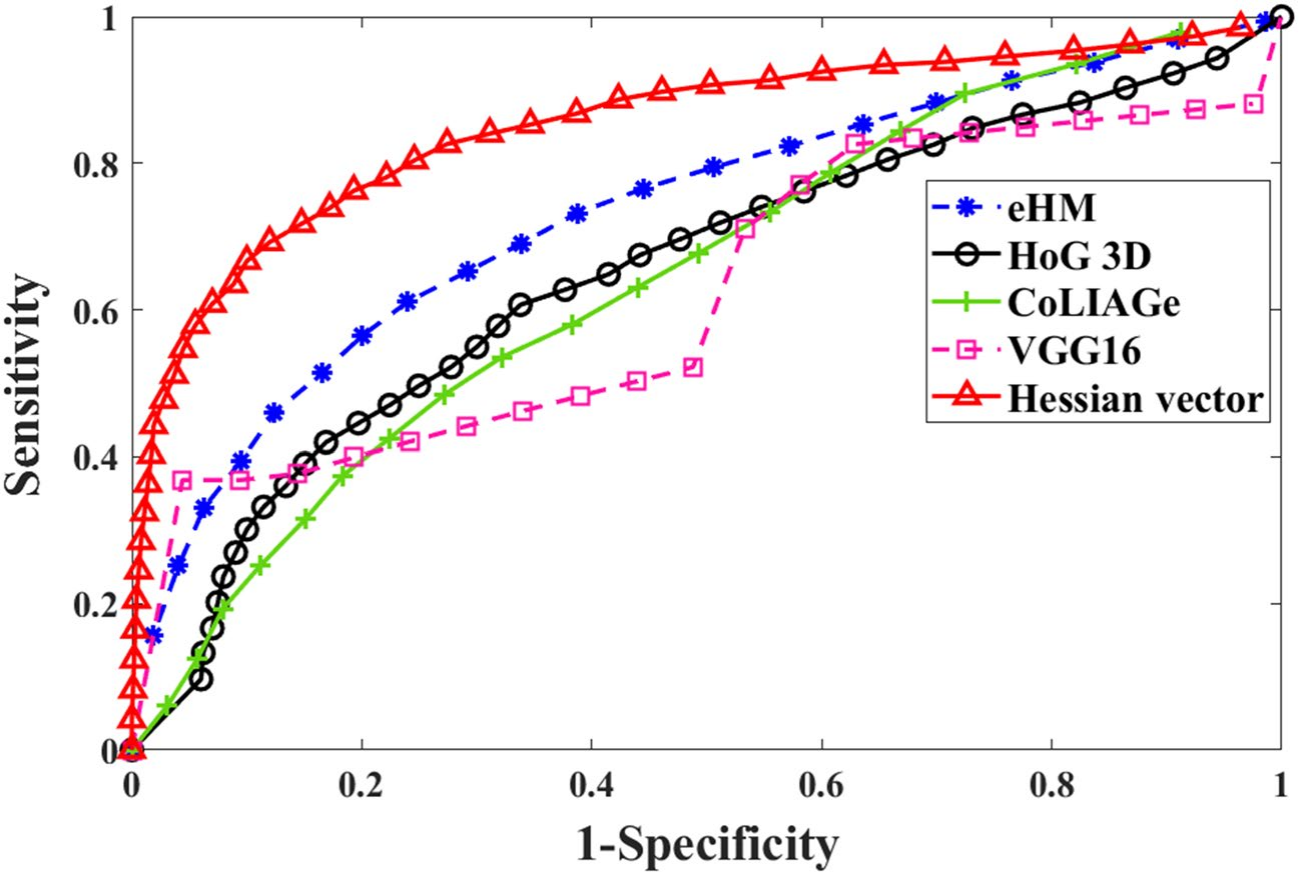
The ROC curves presented for each comparative method and our proposed lesion model over the 68 lung nodules with twofold cross validation.

Figure 4 shows the ROC curves of the Hessian vector representation of our lesion model with comparison to the above four CADx methods over the 68 nodules. The ROC curves in this figure illustrate that VTF based lung nodule classification is much better than eHM, HoG 3D, ColIAGe and VGG16.

Table 4 shows the comparison results among different methods over the 68 lung nodules, where the same RF classifier was used, except for the deep learning VGG16 which performs feature analysis and classification simultaneously. Our proposed lesion model outperformed all the comparison methods, rendering a similar outcome as that from the 63 polyp dataset. When compared to the eHM, our Hessian vector derived texture features outperformed the Haralick texture features with an AUC of 0.861 against 0.792. Compared against the gradient based features, our proposed vector texture features improved the performance substantially over the HoG3D features (AUC = 0.671) and the CoLIAGe texture features (AUC = 0.654). The gain is also substantially higher over the VGG-16 outcome (AUC = 0.669).

**Table 4:** AUC, accuracy, sensitivity, and specificity values over the 68 lung nodules from the comparative methods and our proposed lesion model.

	AUC	Acc	Sen	Spe
eHM	0.792	0.805	0.881	0.631
HoG3D	0.671	0.706	0.496	0.939
CoLIAGe	0.654	0.756	0.930	0.358
VGG16	0.669	0.687	0.673	0.700
Hessian vector	0.861	0.841	0.863	0.786

In addition to the comparisons by the visualization with the ROC plots and the quantitative measures with the four metrics of AUC, Acc, Sen and Spe, we further performed experiments to show the statistical significance among all the CADx methods. Using the Wilcoxin ranked t-test, we obtained the quantitative measures for significant difference between the results of our lesion model or Hessian vector representation and the four reference methods. Table 5 shows the P-values between our Hessian vector model and the four reference methods, indicating that the classification performance of our lesion model is statistically significantly better than the performances of the four reference methods for both the polyp and nodule datasets (p-value ≪ 0.05).

**Table 5:** P-values comparing proposed methods to comparison methods using Wilcoxin ranked sum test over 63 polyps and 68 lung nodules.

Dataset\method	eHM	HoG3D	CoLIAGe	VGG16
63 colon polyps	≪ 0.05	≪ 0.05	≪ 0.05	≪ 0.05
68 lung nodules	≪ 0.05	≪ 0.05	≪ 0.05	≪ 0.05

### Comparison to human observers

Inspired by the competitive game of AlphaGo vs. Expert^30^, we invited three radiologists to score on the above 59 patients’ CTC images, each includes the entire abdomen volume. A total of 63 polyps are embedded inside the 59 CTC images. The human experts are experienced radiologists with 15–35 years of clinical practice in their field. They had all the visualization tools in current clinical setting to exam each polyp, and achieved AUC values of 0.869, 0.926, and 0.960, respectively^39^. Our dynamic lesion model, called AlphaPolyp here, outperformed all three radiologists by AUC score of 0.986. By this comparison study, we have gained the insights below about why both AlphaGo and AlphaPolyp can outperform the human experts.

For the AlphaGo, it not only learns how to play the game, but also knows the task of occupying an area as large as possible. Given a current situation for next move, AlphaGo can make the next move to occupy the largest possible nearby area while human expert may not be able to achieve that task every time. For our AlphaPolyp design, the task-driven AI-enabled algorithm is built on the clinical observations (i.e. the human experts’ learning process) and is trained for the task of matching the pathological outcome of the true lesions. The AlphaPolyp will take all the learnt observations into consideration to predict the pathological outcome while the human experts may not be able to consider all the observations to predict the pathological outcome.

## Conclusions and discussions

A dynamic lesion model was proposed to consider the clinical observation that malignant lesions have a high tendency to invade their surrounding environment compared to benign ones. To mathematically describe the lesion model, one conjecture was made that the lesion growing or invading tendency is related to the rate of image intensity changing at each image voxel, and the rate is represented by the 2nd order derivatives at that voxel, leading to a field of Hessian matrix across the image array space. To circumvent the difficulty of extracting quantitative measures from a matrix field for the task of CADx of lesions, another conjecture was made that the three eigenvalues of the Hessian matrix can be a surrogate representation of the Hessian matrix, and further be treated as a vector in the orthogonal system of the eigenvectors, i.e. Hessian vector.

Inspired by the well-known co-occurrence texture measures for the CADx task, we extracted the co-occurrence vector texture features, i.e. VTFs, from the Hessian vector field or image. Classifying the VTFs for the CADx task on two pathologically proven lesion datasets of polyps and nodules provided striking results, outperforming both the state-of-the-art CADx methods and radiologist experts.

While the VTF was demonstrated as a good feature descriptor of the dynamic lesion model, other vector feature descriptors can be explored for the CADx task. This is one of our future research topics.

While the Hessian vector was demonstrated as a good surrogate of the Hessian matrix, use of the Hessian matrix for generation of matrix feature descriptors for the CADx task is desired. This is another topic of our future research endeavors.

More importantly, by the insights on why AlphaGo can outperform the human experts, we believe task-driven AI-enabled CADx systems would be a direction of our future research endeavors^40–42^.

It can be seen that the evaluation study of the VTF descriptor for the dynamic lesion model is limited by the small sample size of both the polyp and the nodule datasets. We have been devoting significant effort to continually acquire more pathologically proven lesion datasets, although the data acquisition is highly costly.

## Appendix

In our experiments, we choose 15 kinds of total gray levels to compute different **VCM**s, i.e. {16, 24, 32, 40, 48, 56, 64, 72, 80, 88, 96, 104, 112, 120, 128}. According to various of integer factorizations, we generated 235 different combinations of $Q^{a} ,Q^{b} ,{\text{ and}} Q^{c}$ which are listed in Table 6.

**Table 6:** Various of combination groups of Q^c^, Q^a^, and Q^b^ at different total gray levels for Eq. (15) where a gray level is equal to Q^c^ × Q^a^ × Q^b^.

Gray level	The set of (Q^a^, Q^b^, Q^c^)
16	(2, 2, 4), (2, 4, 2), (4, 2, 2)
24	(2, 2, 6), (2, 3, 4), (2, 4, 3), (2, 6, 2), (3, 2, 4), (3, 4, 2), (4, 2, 3), (4, 3, 2), (6, 2, 2)
32	(2, 2, 8), (2, 4, 4), (2, 8, 2), (4, 2, 4), (4, 4, 2), (8, 2, 2)
40	(2, 2, 10), (2, 4, 5), (2, 5, 4), (2, 10, 2), (4, 2, 5), (4, 5, 2), (5, 2, 4), (5, 4, 2), (10, 2, 2)
48	(2, 2, 12), (2, 3, 8), (2, 4, 6), (2, 6, 4), (2, 8, 3), (2, 12, 2), (3, 2, 8), (3, 4, 4), (3, 8, 2), (4, 2, 6), (4, 3, 4), (4, 4, 3), (4, 6, 2), (6, 2, 4), (6, 4, 2), (8, 2, 3), (8, 3, 2), (12, 2, 2)
56	(2, 2, 14), (2, 4, 7), (2, 7, 4), (2, 14, 2), (4, 2, 7), (4, 7, 2), (7, 2, 4), (7, 4, 2), (14, 2, 2)
64	(2, 2, 16), (2, 4, 8), (2, 8, 4), (2, 16, 2), (4, 2, 8), (4, 4, 4), (4, 8, 2), (8, 2, 4), (8, 4, 2), (16, 2, 2)
72	(2, 2, 18), (2, 3, 12), (2, 4, 9), (2, 6, 6), (2, 9, 4), (2, 12, 3), (2, 18, 2), (3, 2, 12), (3, 3, 8), (3, 4, 6), (3, 6, 4), (3, 8, 3), (3, 12, 2), (4, 2, 9), (4, 3, 6), (4, 6, 3), (4, 9, 2), (6, 2, 6), (6, 3, 4), (6, 4, 3), (6, 6, 2), (8, 3, 3), (9, 2, 4), (9, 4, 2), (12, 2, 3), (12, 3, 2), (18, 2, 2)
80	(2, 2, 20), (2, 4, 10), (2, 5, 8), (2, 8, 5), (2, 10, 4), (2, 20, 2), (4, 2, 10), (4, 4, 5), (4, 5, 4), (4, 10, 2), (5, 2, 8), (5, 4, 4), (5, 8, 2), (8, 2, 5), (8, 5, 2), (10, 2, 4), (10, 4, 2), (20, 2, 2)
88	(2, 2, 22), (2, 4, 11), (2, 11, 4),(2, 22, 2), (4, 2, 11), (4, 11, 2), (11, 2, 4), (11, 4, 2), (22, 2, 2)
96	(2, 2, 24), (2, 3, 16), (2, 4, 12), (2, 6, 8), (2, 8, 6), (2, 12, 4), (2, 16, 3), (2, 24, 2), (3, 2, 16), (3, 4, 8), (3, 8, 4), (3, 16, 2), (4, 2, 12), (4, 3, 8), (4, 4, 6), (4, 6, 4), (4, 8, 3), (4, 12, 2), (6, 2, 8), (6, 4, 4), (6, 8, 2), (8, 2, 6), (8, 3, 4), (8, 4, 3), (8,6, 2), (12, 2, 4), (12, 4, 2), (16, 2, 3), (16, 3, 2), (24, 2, 2)
104	(2, 2, 26), (2, 4, 13),(2, 13, 4), (2, 26, 2), (4, 2, 13), (4, 13, 2), (13, 2, 4), (13, 4, 2), (26, 2, 2)
112	(2, 2, 28), (2, 4, 14), (2, 7, 8), (2, 8, 7), (2, 14, 4), (2, 28, 2), (4, 2, 14), (4, 4, 7), (4, 7, 4), (4, 14, 2), (7, 2, 8), (7, 4, 4), (7, 8, 2), (8, 2, 7), (8, 7, 2), (14, 2, 4), (14, 4, 2), (28, 2, 2)
120	(2, 2, 30), (2, 3, 20), (2, 4, 15), (2, 5, 12), (2, 6, 10), (2, 10, 6), (2, 12, 5), (2, 15, 4), (2, 20, 3), (2, 30, 2), (3, 2, 20), (3, 4, 10), (3, 5, 8), (3, 8, 5), (3, 10, 4), (3, 20, 2), (4, 2, 15), (4, 3, 10), (4, 5, 6), (4, 6, 5), (4, 10, 3), (4, 15, 2), (5, 2, 12), (5, 3, 8), (5, 4, 6), (5, 6, 4), (5, 8, 3), (5, 12, 2), (6, 2, 10), (6, 4, 5), (6, 5, 4), (6, 10, 2), (8, 3, 5), (8, 5, 3), (10, 2, 6), (10, 3, 4), (10, 4, 3), (10, 6, 2), (12, 2, 5), (12, 5, 2), (15, 2, 4), (15, 4, 2), (20, 2, 3), (20, 3, 2), (30, 2, 2)
128	(2, 2, 32), (2, 4, 16), (2, 8, 8), (2, 16, 4), (2, 32, 2), (4, 2, 16), (4, 4, 8), (4, 8, 4), (4, 16, 2), (8, 2, 8), (8, 4, 4), (8, 8, 2), (16, 2, 4), (16, 4, 2), (32, 2, 2)
